# Decreased HHV-6 IgG in Alzheimer’s Disease

**DOI:** 10.3389/fneur.2017.00040

**Published:** 2017-02-20

**Authors:** Gabriel Westman, Jonas Blomberg, Zhibing Yun, Lars Lannfelt, Martin Ingelsson, Britt-Marie Eriksson

**Affiliations:** ^1^Department of Medical Sciences, Uppsala University, Uppsala, Sweden; ^2^Department of Laboratory Medicine, Karolinska Institutet, Stockholm, Sweden; ^3^Department of Public Health and Caring Sciences, Uppsala University, Uppsala, Sweden

**Keywords:** Alzheimer’s disease, herpesvirus, HHV-6, multiplex, immunoassay, IgG

## Abstract

Human herpesviruses have previously been implicated in the pathogenesis of Alzheimer’s disease (AD) but whether they are causal, facilitating, or confounding factors is yet to be established. A total of 50 AD subjects and 52 non-demented (ND) controls were analyzed in a multiplex assay for IgG reactivity toward herpes simplex virus (HSV), varicella zoster virus (VZV), cytomegalovirus (CMV), and human herpesvirus 6 (HHV-6). The HHV-6 IgG reactivity was significantly lower in AD subjects compared to ND controls, whereas there were no differences in HSV, VZV, or CMV antibody levels between the groups. Analysis of peripheral blood mononuclear cells with a subtype-specific HHV-6 PCR revealed no signs of reactivation, as AD and ND subjects presented with comparable HHV-6 DNA levels in PBMCs, and all positive samples were of subtype B. Whether HHV-6 is a factor in AD remains to be elucidated in future studies.

## Introduction

It has been debated whether infectious agents are causing, triggering, or facilitating the most common age-related diseases, such as cardiovascular disease, dementia, diabetes, and cancer. Well-proven examples include human papillomavirus, causing the majority of cervical cancers, and *Helicobacter pylori*, causing duodenal ulceration (1, 2), but several other pathogen-disease links have been suggested where data are conflicting or controversial.

Human herpesviruses are widely spread in the population, with the seroprevalence of varicella zoster virus (VZV), Epstein–Barr virus (EBV), cytomegalovirus (CMV), and human herpesvirus 6 (HHV-6) increasing rapidly in the first years of life, whereas herpes simplex virus (HSV) infection generally occurs at more advanced ages (3–6). HSV types 1 and 2, VZV, and HHV-6A are all neurotropic and can cause acute central nervous system (CNS) illness even in immunocompetent hosts (7–10). CMV is less prone to infect neurons but target endothelial cells, microglia, and hematopoietic cells, including those that reside in the brain and could therefore have indirect effects through immune modulation or inflammatory mediators (11).

Alzheimer’s disease (AD), with approximately 36.5 million people currently affected worldwide, is the most common form of dementia, causing substantial suffering for patients and a heavy burden on caretakers (12). Whether infectious diseases can be causal, facilitating, or confounding factors in the pathogenesis of AD is yet to be established. Studies of AD *postmortem* brain tissue have shown an increased prevalence of HSV-1 DNA in patients carrying the *ApoE* ϵ*4* allele and HHV-6 DNA regardless of *ApoE* genotype (8).

In terms of humoral immune response, it has been shown that serum levels of HSV IgM, but not IgG, antibodies correlates with AD (13–15). Furthermore, it has been suggested that there is a cumulative correlation between a serologic pattern of multiple herpesvirus infections and cognitive decline in patients with vascular disease (16, 17). Also, a connection between CMV and AD was made in a combined clinical and postmortem study where CMV IgG levels and IFN-γ in cerebrospinal fluid correlated with neuropathologic characteristics of AD (18). This is consistent with the findings in a previous prospective study, where an increased rate of cognitive decline was seen in patients with high levels of CMV IgG (19).

We have previously shown that AD patients present with a lower frequency of CMV-specific CD8+ T-cells and that CMV-seropositive AD patients show a more pro-inflammatory peripheral blood mononuclear cell (PBMC) phenotype than both CMV-seronegative AD patients and CMV-seropositive non-demented (ND) controls (20, 21). In this study, we have investigated whether patients with AD present with a different herpesvirus-specific serologic pattern compared to ND controls, using an in-house multiplex IgG immunoassay with antigens generated by lysis of purified virions from infected cells. Furthermore, we have investigated whether the differences seen in the serologic reactivity pattern could be verified by PCR analysis of PBMCs.

## Materials and Methods

### Subjects and Sampling Procedure

A total of 51 AD patients and 52 ND controls were included from a previously described cohort (20). One participant in the AD group was excluded due to a change in the diagnosis from AD to frontotemporal dementia, rendering a total of 50 AD subjects and 52 ND controls. Demographics and cognitive performance of the study groups is described in Table 1. All AD subjects had recently received a clinical AD diagnosis in accordance with the NINCDS-ADRDA criteria and DSM-IV criteria. Thus, all patients described a clinical picture of AD and a CT or MRI scan consistent with the diagnosis, i.e., without significant vascular abnormalities. Subjects in the ND control group had been recruited *via* local advertising and did not show any frank clinical signs that could suggest the presence of cognitive deficits or complain of any subjective cognitive impairment. Blood samples were acquired by venipuncture, performed at the Memory Disorder Unit at Uppsala University Hospital. Plasma was separated from blood cells through centrifugation, and the samples were frozen at −20°C until analyzed. PBMCs were isolated using BD Vacutainer CPT™ Cell Preparation Tubes with sodium citrate and frozen in batches of 5 × 10^6^ cells in medium consisting of 15% dimethyl sulfoxide and 85% fetal calf serum. The dementia status of all study participants were blinded during the laboratory work.

**Table 1:** **Summary of baseline characteristics in patients with Alzheimer’s disease (AD) and non-demented controls (ND)**.

Continuous data reported as mean (SD)	AD (*N* = 50)	ND (*N* = 52)
Age, years	77.5 (6.9)	74.2 (7.9)
Gender, male/female	28/22	23/29
Mini-Mental State Examination score	19.9 (4.8)	NA
*APOE* ϵ4 allele carriers, hetero-/homozygote	28/4	16/2

*NA, not available*.

This study was carried out in accordance with the Declaration of Helsinki. Written informed consent was obtained from all study participants, together with consent from a close relative if the subject was considered incapable of taking his or her own decision. The study was approved (no. 2009/097) by the Regional Ethical Review Board in Uppsala, Sweden.

### Antigens

The following antigens (Advanced Biotechnologies, Eldersburg, MD, USA) were used for the analyses: herpes simplex type 1 (strain MacIntyre, cat. no. 10-145-000, purified by ultracentrifugation, control antigen: VERO cells, cat. no. 10-508-001), CMV (strain AD169 purified viral lysate, cat. no. 10-144-000, control antigen: human foreskin fibroblasts cells, cat. no. 10-505-001), VZV (strain rod, pelleted virus from supernatant from an infected culture, cat. no. 10-282-500, control antigen: VERO cells), and HHV-6A (GS strain, pelleted virus from supernatant of an infected culture, cat. no. 10-241-500, control antigen: HSB-2 cells, cat. no. 10-529-001). The purified virions were lysed by exposure to the non-ionic detergent Triton-X100 (1% w/v) for 10 min at room temperature. Antigens were dialyzed overnight against phosphate buffered saline before coupling. Coupling to beads at lysine positions of the peptides were made using water-soluble carbodiimide according to the procedure described by Luminex Corporation. Between 10 and 30 µg of protein was used per coupling, which sufficed for approximately 1,000 tests.

### Multiplex Immunoassay

All antigens were covalently coupled to carboxylated color-coded beads, as previously described by Elfaitouri et al (22). IgG was detected using biotinylated protein G (23). The specificity of the antibody detection was confirmed by using 4 µg/ml biotinylated monoclonal anti-IgG (BioLegend, San Diego, CA, USA, cat. no. 409307). Results are expressed as median fluorescent intensity. StabilGuard (SurModics, Eden Prairie, MN, USA) was used as a sample diluent throughout the process. Samples were diluted 1:10, including the preparation steps prior to loading the filter plate. A final bead concentration of 25 beads/μl was used during analysis. All samples and controls were sonicated and vortexed for 20 s before being added to the bead mixture.

The standard Luminex protocol for indirect antibody capture immunoassay was followed with the previously described adaptations (22). Samples (100 µl) were analyzed on a Luminex^®^ 200™ system, in accordance with the manufacturer’s instructions. The StarStation (Applied Cytometry, Sheffield, UK) and xPONENT^®^ (Luminex Corporation, Austin, TX, USA) software were used to analyze the data.

### HHV-6 PCR

In brief, HHV-6 DNA was extracted from 200,000 PBMCs using MagNA Pure LC Total Nucleic Acid Isolation Kit—High Performance (Roche Diagnostics). HHV-6 DNA quantification was performed using a RealStar HHV-6 PCR kit 1.0 (Altona Diagnostics) on an ABI 7500 FAST Real-time PCR instrument (Applied Biosystems). The PCR program consisted of 1 cycle at 95°C in 10 min followed by 45 cycles at 95°C in 15 s and 58°C in 1 min. To evaluate potential PCR inhibition, an internal control was included in each PCR and detected with a JOE-labeled IC-specific probe. The PCR method differentiates HHV-6A and HHV-6B through a FAM-labeled HHV-6A and a Cy5-labeled HHV-6B-specific probe. From the specification provided by the PCR kit manufacturer, the analytical sensitivity of the PCR assay was calculated to two copies of HHV-6 DNA, but the primer and probe sequences are not given. Subtype specificity of the assay was confirmed by testing cultured HHV-6A GS Strain and HHV6B Z-29 Strain. The specificity of the assay was further verified by testing HSV-1, HSV-2, VZV, CMV, EBV, HHV-7, HHV-8, BK, and JC virus, parvovirus, hepatitis A, B, C, and HIV-1.

### Statistical Analysis

R version 3.0.2 (The R Foundation for Statistical Computing) with packages *coin* (version 1.1.0) and *latticeExtra* (version 0.6.26) was used for statistical analysis and graphical output. AD and ND groups were compared using a multivariate Mann–Whitney test. Multiplicity adjustment was done using a permutation-based step-down procedure accounting for the correlation between the test statistics (24).

## Results

### Multiplex Immunoassay

**Figure 1:**
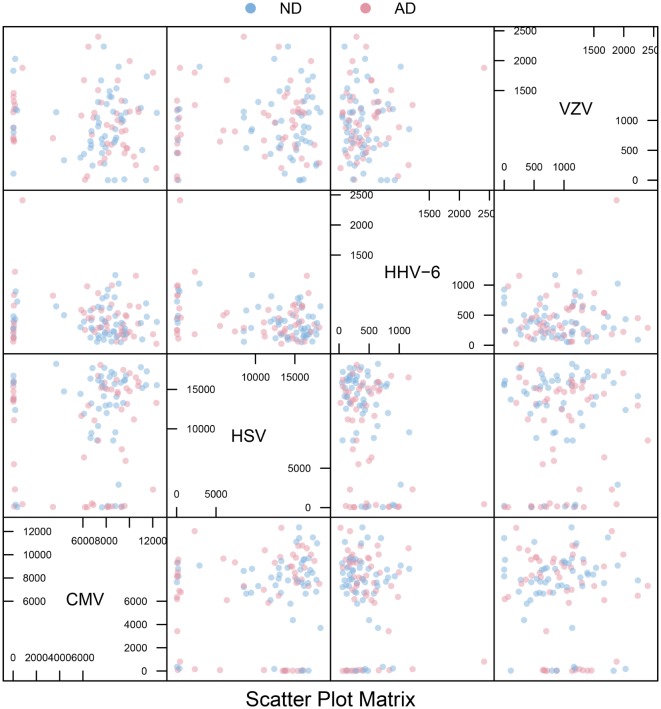
**Scatter plot matrix**. Pairwise plots for all combinations of antigens, comparing antibody distributions for Alzheimer’s disease (AD) and non-demented (ND) groups. The overall absence of strong diagonal patterns indicates no obvious cross-reactivity between antibodies.

**Figure 2:**
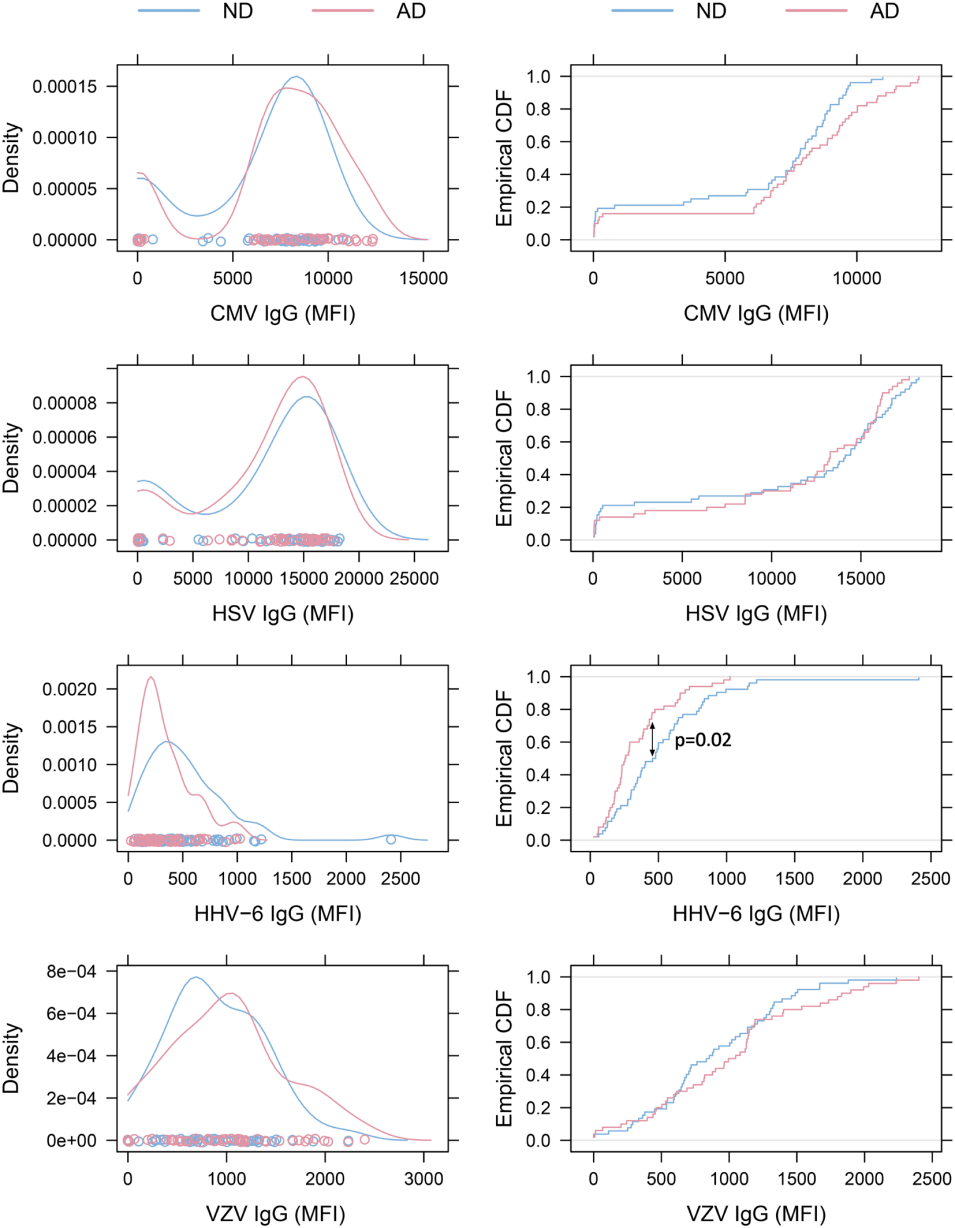
**Density plots and empirical cumulative distribution functions (ECDF)**. Left: density plots of virus-specific antibody levels. The distributions for cytomegalovirus (CMV) and herpes simplex virus (HSV)-1 illustrate clustering of subjects into seronegative and seropositive populations. No obvious clustering was seen in human herpesvirus 6 (HHV-6) and varicella zoster virus (VZV) plots, as expected when seroprevalence is close to 100%. Right: ECDF plots illustrating that Alzheimer’s disease (AD) patients present with lower HHV-6 antibody levels than non-demented (ND) controls (*p* = 0.02 with Bonferroni-Holm correction).

All IgG reactivity data were plotted in a matrix of pairwise comparisons, revealing no obvious patterns of cross-reactivity between the respective sets of antigens (Figure 1). Density plots (Figure 2) showed clustering into seronegative and seropositive populations for CMV and HSV-1. For HHV-6 and VZV, there was no clear separation into subpopulations, most likely due to a very high seroprevalence in the study population.

**Figure 3:**
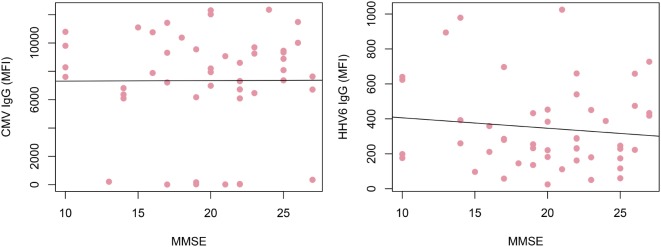
**Correlation of IgG reactivity and cognitive performance in Alzheimer’s disease (AD) patients**. No correlation could be found between cytomegalovirus (CMV) or human herpesvirus 6 (HHV-6) IgG levels and MMSE score. Only AD patients were included.

Alzheimer’s disease patients presented with significantly lower HHV-6 IgG reactivity compared to ND controls (*p* = 0.02 after multiplicity adjustment, Figure 2). The numerical difference in CMV IgG between AD and ND groups failed to reach statistical significance (*p* = 0.28 after multiplicity adjustment). Within the AD group, there was no correlation between MMSE score and IgG levels for HHV-6 or CMV (Figure 3). Neither were there any significant differences between AD and ND groups with respect to HSV or VZV IgG levels. Both positive and negative results were verified in a logistic regression model including all antibody levels, age, and gender.

**Figure 4:**
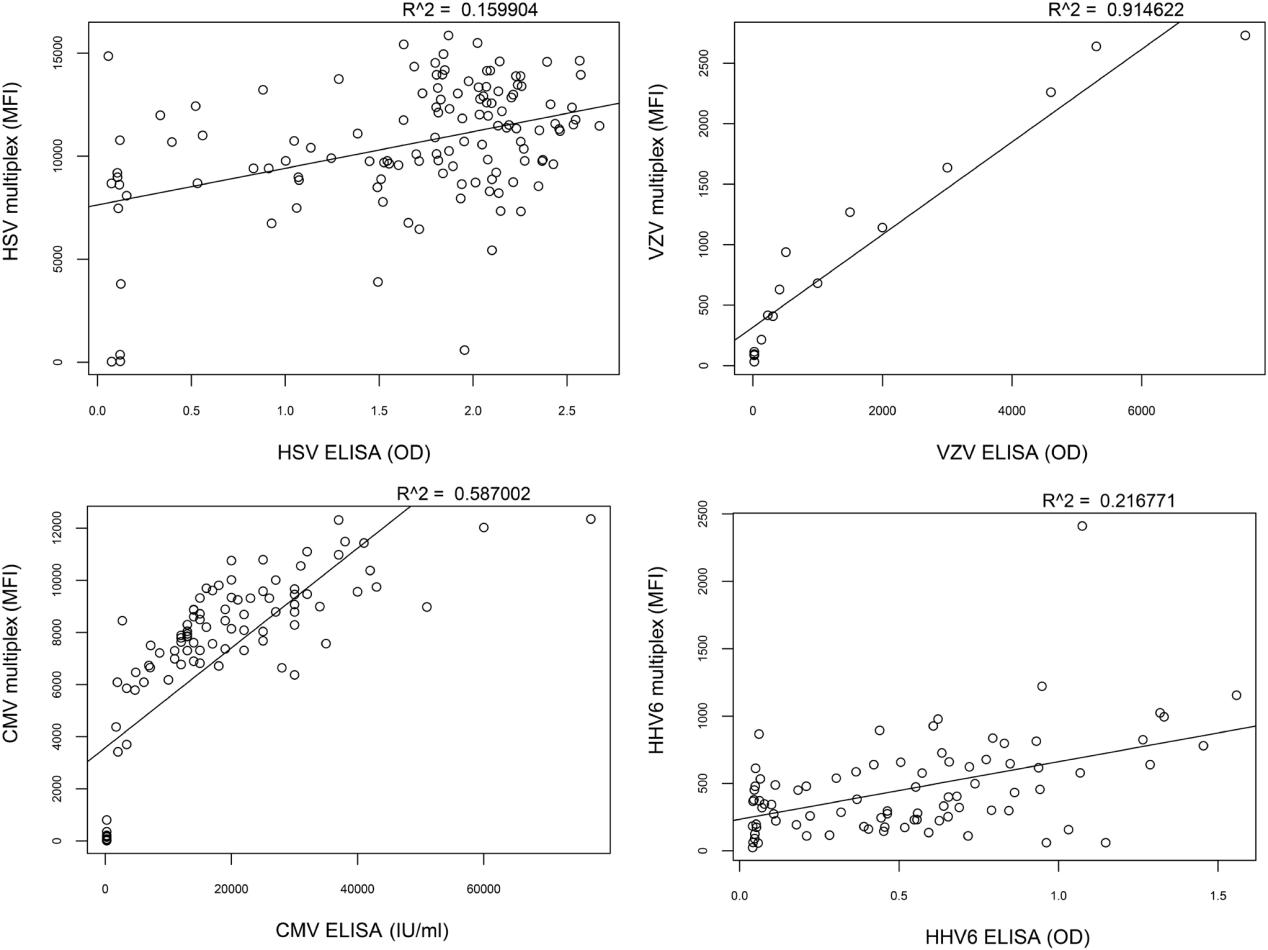
**Correlation between multiplex assay and reference methods**. The correlation of IgG reactivity between the multiplex assay and standard ELISA is low for herpes simplex virus (HSV) (*R*^2^ = 0.16) and human herpesvirus 6 (HHV-6) (*R*^2^ = 0.22), intermediate for cytomegalovirus (CMV) (*R*^2^ = 0.59), and high for varicella zoster virus (VZV) (*R*^2^ = 0.91).

To assess the correlation between the whole-virus IgG reactivity in the multiplex assay and microbiologic reference methods, a selected set of samples was also analyzed using single-agent ELISA kits (Focus Diagnostics HerpeSelect HSV IgG, Vidas CMV IgG, Mobitech anti-HHV-6 IgG, and Siemens Enzygnost anti-VZV/IgG). The epitopes created through the in-house lysis of purified virions in the multiplex assay resulted in differences compared to reference methods and illustrated that the assays are not directly interchangeable (Figure 4).

### HHV-6 PCR

As the multiplex immunoassay indicated a possible difference in HHV-6 humoral immunity between AD and ND groups, we proceeded to analyze PBMC samples with a subtype-specific quantitative HHV-6 PCR to establish whether there was a difference in peripheral viral reactivation between groups. A total of 50 AD and 52 ND subjects were analyzed, rendering 4 HHV-6 DNA-positive results in the AD group (8%) and 4 in the ND group (7.7%). All positive samples were of HHV-6 subtype B. There was no clear difference in DNA levels between the two groups (Table 2). Two AD and one ND participants presented with DNA levels in the 10^6^ copies (per 10^6^ cells) range, most likely related to chromosomally integrated HHV-6.

**Table 2:** **Subtype-specific HHV-6 PCR**.

Gender	Age	Dementia status	HHV-6 DNA	Subtype
Male	68	Alzheimer’s disease (AD)	4 × 10^6^	B
Male	76	AD	2 × 10^6^	B
Male	80	AD	9 × 10^3^	B
Female	72	AD	8 × 10^1^	B
Female	72	Non-demented (ND)	6 × 10^1^	B
Male	51	ND	2 × 10^6^	B
Male	75	ND	1 × 10^2^	B
Female	75	ND	2 × 10^4^	B

*Overview of AD and ND subjects positive for HHV-6 DNA in PBMCs. DNA levels are expressed as copies per million cells*.

## Discussion

In this study, we illustrate the application of a multiplex immunoassay with in-house-generated antigens to compare IgG reactivity against four human herpesviruses of which some have previously been connected to AD, both seroepidemiologically (13–17) and by the presence of viral DNA in AD brain tissue (25). Although the assay would need additional validation to be useful in a clinical setting for the diagnosis of herpesvirus infections, we believe the current results are valid for the within-study comparison of humoral herpesvirus immunity between AD and ND subjects. Moreover, this alternative methodological approach could provide new insights in addition to the previous studies performed with regular single ELISA assays.

The finding that AD subjects have significantly lower levels of HHV-6 IgG reactivity compared to ND controls is in contrast to the previous findings by Agostini et al., who did not see a correlation between HHV-6 IgG and cognitive performance (26). Also, the fact that demented subjects were found to present with lower IgG toward an agent in the herpesvirus group is different from what is previously known from studies of CMV and HSV-1, where cognitive decline has been associated with higher antibody levels (14, 15, 19). However, as cell tropism differs within the herpesvirus group, the humoral immune response could have different effects on the AD pathophysiology depending on the type of virus and possibly also the stage of disease.

These findings, suggesting an impaired humoral immunity against HHV-6, could provide an explanation to the more frequent findings of HHV-6 DNA in AD brain tissue, although this study does not give any evidence of causality. The work by Carbone et al. could however give a hint, as they observed an increased risk of progression to AD in the subjects in the control group that presented with a positive HHV6 PCR on peripheral blood at baseline (25). With an expected seroprevalence of HHV-6 close to 100% in these age groups, the differences are probably not attributable to total prevalence of chronically persistent HHV-6 infection but rather to differences in humoral immune response. One could speculate on whether decreased antibody levels increase the risk of local CNS HHV-6 reactivation, elevating the inflammatory response that could enhance AD pathology (27). Other possible explanations include age differences between AD and ND groups at the time of primary infection or more profound immunologic changes in AD affecting herpesvirus immunity in general.

As there was no difference in HHV-6 DNA in PBMC between the groups, and all positive samples were of subtype B, the results do not support the hypothesis that peripheral reactivation of HHV-6 of any subtype is implicated in AD pathogenesis. However, as we did not analyze brain tissue samples, we cannot exclude local HHV-6 reactivation. These results are in conflict with a previous study by Carbone et al. who found HHV-6 DNA five times more frequently in peripheral blood leukocytes from AD subjects compared to controls (25).

Array-based multiplex techniques have made it possible to measure antibodies to many infectious agents simultaneously with a small sample volume, allowing a rapid syndrome-centered overview of pathogen antibodies, and provide better control of false-positive reactions due to cross-reactivity than what can be achieved with single assays. It has been previously reported that multiplexing serologic assays can introduce negative matrix effects caused by interindividual variations in serum lipids, proteins, heterophilic antibodies, and immune complexes (28, 29). However, the protocol used in this study uses a lower concentration of carrier proteins and has so far proven robust. We chose to measure only IgG reactivity as the relevance of IgM, especially outside the clinical context of paired sampling in primary infection, which is limited not only by the methodological issues mentioned above but also by the natural cross-reactivity between different members of the herpesvirus group due to their structural similarity that affects IgM more than IgG (30, 31).

As the multiplex serologic protocol used in this study has not been extensively validated against the gold standard methods used in clinical practice, any comparison with these should be made with caution. Also, the serologic distinction between HHV-6A and HHV-6B is difficult to make due to cross-reactivity, so the identified differences are likely to be driven by a large contribution of HHV6-B antibodies.

Although several other members of the human herpesvirus family have been previously correlated with cognitive decline, we could not—with the exception of HHV-6—find any serological evidence supporting the hypothesis that infections with HSV, VZV, or CMV correlate with AD. The precision of these negative results is, however, limited by the sample size in this study. Whether HHV-6 is involved in the pathogenesis of AD or other neurodegenerative diseases remains to be elucidated in future studies.

## Author Contributions

GW, B-ME, JB, MI, and LL—substantial contributions to the conception or design of the work. GW, JB, B-ME, ZY, and MI—acquisition, analysis, or interpretation of data for the work. All authors—drafting the work or revising it critically for important intellectual content. All authors—final approval of the version to be published. All authors—agreement to be accountable for all aspects of the work in ensuring that questions related to the accuracy or integrity of any part of the work are appropriately investigated and resolved.

## Conflict of Interest Statement

The authors declare that the research was conducted in the absence of any commercial or financial relationships that could be construed as a potential conflict of interest.
